# A Proteomic Analysis of Individual and Gender Variations in Normal Human Urine and Cerebrospinal Fluid Using iTRAQ Quantification

**DOI:** 10.1371/journal.pone.0133270

**Published:** 2015-07-29

**Authors:** Zhengguang Guo, Yang Zhang, Lili Zou, Danqi Wang, Chen Shao, Yajie Wang, Wei Sun, Liwei Zhang

**Affiliations:** 1 Core Facility of Instrument, Institute of Basic Medical Sciences, Chinese Academy of Medical Sciences, School of Basic Medicine, Peking Union Medical College, 5 Dong Dan San Tiao, Beijing, China, 100005; 2 Department of Neurosurgery/China National Clinical Research Center for Neurological Diseases, Beijing Tiantan Hospital, Capital Medical University, 6 Tian Tan Xi Li, Beijing, China, 100050; 3 National Key Laboratory of Medical Molecular Biology, Department of Physiology and Pathophysiology, Institute of Basic Medical Sciences, Chinese Academy of Medical Sciences, School of Basic Medicine, Peking Union Medical College, 5 Dong Dan San Tiao, Beijing, China, 100005; 4 Core Laboratory for Clinical Medical Research, Beijing Tiantan Hospital, Capital Medical University, 6 Tian Tan Xi Li, Beijing, China, 100050; 5 Department of Clinical Laboratory Diagnosis, Beijing Tiantan Hospital, Capital Medical University, 6 Tian Tan Xi Li, Beijing, China, 100050; University of Nebraska Medical Center, UNITED STATES

## Abstract

Urine and cerebrospinal fluid (CSF) are two important biofluids used for disease biomarker discovery. For differential proteomic analysis, it is essential to evaluate individual and gender variations. In this study, we characterized urinary and CSF proteomes of 14 healthy volunteers with regard to individual and gender variations using 2DLC-MS/MS analysis and 8-plex iTRAQ quantification. A total of 968/512 urinary/CSF proteins were identified, with 406/280 quantified in all individuals. The median inter-individual coefficients of variation (CVs) were 0.262 and 0.183 for urinary and CSF proteomes, respectively. Cluster analysis showed that male and female urinary proteomes exhibited different patterns, though CSF proteome showed no remarkable gender differences. In comparison with CSF proteome, urinary proteome showed higher individual variation. Further analysis revealed that individual variation was not correlated with protein abundance. The minimum sample size for proteomic analysis with a 2-fold change was 10 (4/5 for males/females using iTRAQ quantification) for urinary or 8 for CSF proteome. Intracellular proteins leaked from exfoliative cells tended to have higher CVs, and extracellular proteins secreted from urinary tract or originating from plasma tended to have lower CVs. The above results might be beneficial for differential proteomic analysis and biomarker discovery.

## Introduction

Biofluids, which include plasma, urine, cerebrospinal fluid (CSF), saliva, and skin suction blister fluid, contain various proteins that can provide useful information about human conditions. With the development of proteomic technologies, biofluid proteome has become an important research field. Urine and CSF are two important biofluids. Indeed, urinary proteome contains useful information about kidneys, urinary tract and other organs [1,2], and urinary proteome has been used to identify biomarkers of bladder cancer [3], chronic kidney disease [4], and diabetic nephropathy [5]. CSF can provide information about central nervous system (CNS), and this fluid has been used for the analysis of a number of CNS diseases, including Alzheimer’s disease (AD) [6,7], Parkinson’s disease [8], and pediatric medulloblastoma [9].

Before applying proteomic methods to search urinary or CSF biomarkers, a central question to answer is how large of the variations in the normal urine or CSF proteome. Several publications have reported above issues. As early as 2006, Thongboonkerd et al. [10] first compared urinary samples from four men and four women by two-dimensional gel electrophoresis (2-DE); these authors observed the existence of inter-individual variation of the urinary proteome, and they validated both intra- and inter-individual variations in albumin and transferrin. Liu et al.[1] analyzed urinary proteome variations among samples from 10 males and 10 females by 1DLC-MS and the spectral count (SC) method, observing inter-individual variations in nearly 100 proteins (median CV = 0.71). Using 1DLC-MS/MS and label-free method, Nagaraj et al.[11] analyzed seven healthy volunteers (5 males and 2 females). The authors quantified more than 500 proteins in all samples, and the medians for technical, intra- and inter-individual variances were 0.18, 0.48 and 0.66, respectively.

In 2005, Yan Hu et al. first analyzed CSF samples (12 in total) composed of 6 pairs from six individuals obtained 2 weeks apart. Using multi-affinity depletion and two-dimension differential in-gel electrophoresis (2D-DIGE) methods, the researchers analyzed intra- and inter-individual variations in several proteins, with 23 proteins showing a greater than 1.5-fold change within individuals over the 2-week period [12]. Marcel et al. [13] compared CSF proteomes from 10 individuals without neurological diseases using 1D-LC-MS and label-free quantification, and the median technical and inter-individual variations of 126 commonly identified proteins were 0.24 and 0.42, respectively. Steven E. Schutzer et al. [14] compared intra-individual variations and inter-individual variations in 10 volunteer subjects at least 4 weeks apart and found that compared to inter-individual variation, there was little variability in CSF proteins within a subject. In 2013, Richard J. Perrin et al. [15] compared CSF proteome in two aliquots from six cognitively normal individuals using 1DLC-MS and label-free quantification; the median subject variance of 81 quantified proteins was 0.55.

Although inter-individual variations have been studied in several laboratories, there are still important questions that require further investigation. In terms of biological prospects, the inter-individual variation in different genders and in different biofluids needs to be further clarified. Regarding technical prospects, individual variations have primarily been analyzed by 1DLC/MS/MS and label-free method. In recent years, 2D-LC/MS/MS and isobaric tags for the relative and absolute quantitation (iTRAQ) method have been widely used for differential proteomic study, but individual variations have not yet been evaluated by iTRAQ labeling method.

**Fig 1:**
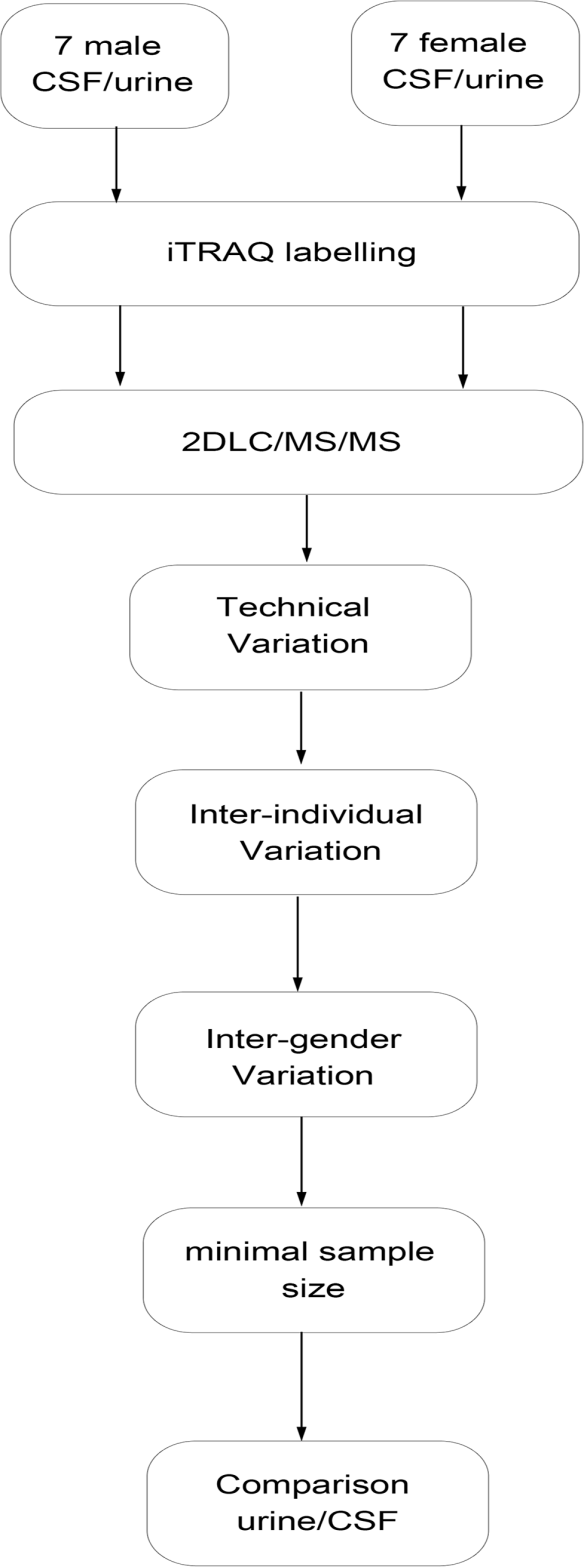
Workflow of the quantitative analysis of individual variation in normal human urinary and cerebrospinal fluid (CSF) proteomes.

In this study, we analyzed inter-individual and inter-gender variations in urinary and CSF proteomes by 2DLC/MS/MS and iTRAQ method. The second morning urine and CSF from 7 healthy male and 7 healthy female volunteers were used to evaluate variations in these urine and CSF proteomes. Each urinary or CSF sample was labeled with 8-plex iTRAQ regents and analyzed by 2DLC MS/MS; pooled urinary and CSF samples were used as controls. Technical variations, inter-individual variations, and inter-gender variations were analyzed. The minimum sample size needed for the urinary and CSF proteomes was evaluated for differential analyses. The difference between individual variation in urine and individual variation in CSF was also analyzed (Fig 1).

## Materials and Methods

### Reagents and instruments

HPLC-grade acetonitrile (ACN), formic acid, trifluoroacetic acid, ammonium bicarbonate, iodoacetamide (IAA), and dithiothreitol (DTT) were purchased from Sigma (St. Louis, MO, USA). Sequencing-grade trypsin was purchased from Promega.

A TripleTOF 5600 mass spectrometer from ABsciex (Framingham, MA, USA) and an ACQUITY UPLC system from Waters (Milford, MA, USA) were used.

### Sample preparation

Second morning urine samples were obtained from 7 healthy males and 7 healthy females (age 23–35 years, with a median age of 27 years) (detailed data are presented in Table A in S1 File). The urinary samples were centrifuged at 5000 x g for 30 min, and the precipitates were removed. The supernatants were precipitated overnight at -4°C using 3 times the volume of ethanol. After 30 min of centrifugation at 10000 x *g*, the pellets were resuspended in lysis buffer (7 M urea, 2 M thiourea, 0.1 M DTT, and 50 mM Tris).

CSF samples were collected by lumbar puncture from patients who received spinal anesthesia before non-neurological operations in Beijing Tiantan Hospital. These patients were assessed by an independent medical doctor to rule out neurological diseases and to determine recent medication history. Following collection, a subsample of each CSF sample was sent to a clinical laboratory for routine CSF diagnostics. The remaining portion of the sample was immediately centrifuged for 10 min at 2500 x *g* to remove cellular components and then stored at -80°C for further analysis. Samples from a total of 7 male and 7 female neurologically intact individuals (aged 24 to 55 years, with a median age of 28 years) were used (detailed data in Table A in S1 File). All selected samples had normal clinical laboratory values with respect to microbiology, chemistry, and cell counts.

Approval for this study was obtained from the review boards in accordance with ethics regulations. All clinical investigations were conducted according to the principles expressed in the Declaration of Helsinki. Our study was approved by the Neurosurgical Clinical Information and Biobanking Project of Beijing Tiantan Hospital (Brain Tumor Section) (NCIBP) (Ethics Number: KY2014-021-02). The participants provided their written informed consent to participate in this study, and the ethics committees/IRBs approved the procedure used to obtain consent.

### Sample preparation

Each sample was digested using filter-aided sample preparation (FASP) method described by R Wisniewsk et al. [16]. Protein samples (200 μg) were reduced with 20 mM DTT at 37°C for 1 h and then carboxyamidomethylated with 50 mM IAA at room temperature in the dark for 45 min. Trypsin (4 μg) in 25 mM NH_4_HCO_3_ was added to protein samples for digestion overnight at 37°C. The resulting peptides were mixed equally to create an internal standard and labeled with the 113 iTRAQ regent. The 7 male/female samples were individually labeled with 114, 115, 116, 117, 118, 119 and 121 iTRAQ regents according to the manufacturer’s protocol (ABsciex). Finally, four pooled samples (male urine, female urine, male CSF, and female CSF) were analyzed by 2DLC/MS/MS.

### HPLC

The pooled mixture of labeled samples was fractionated using a high-pH RPLC column from Waters (4.6 mm×250 mm, Xbridge C18, 3 μm). The samples were loaded onto the column in buffer A2 (H2O, pH = 10). The elution gradient was 5–30% buffer B2 (90% ACN, pH = 10; flow rate = 1 mL/min) for 60 min. The eluted peptides were collected at a rate of one fraction per minute, and the 60 dried fractions were re-suspended in 0.1% formic acid and pooled into 10 samples by combining fractions 1, 11, 21, 31, 41, 51; 2, 12, 22, 32, 42, 52; and so on. A total of 40 fractions from the urinary and CSF peptide mixtures (male urine, female urine, male CSF and female CSF) were analyzed by LC-MS/MS.

### LC/MS/MS

Each fraction was analyzed using a reverse-phase C18 self-packed capillary LC column (75 μm×100 mm, 3 μm). The elution gradient was 5–30% buffer B1 (0.1% formic acid, 99.9% ACN; flow rate, 0.3 μL/min) for 40 min. A TripleTOF 5600 mass spectrometer was used to analyze the eluted peptides from LC, and each fraction was run three times. The MS data were acquired under high-sensitivity mode using the following parameters: 30 data-dependent MS/MS scans per full scan, full scans acquired at a resolution of 40,000 and MS/MS scans at a resolution of 20,000, rolling collision energy, charge-state screening (including precursors with a charge state of +2 to +4), dynamic exclusion (exclusion duration 15 s), an MS/MS scan range of 100–1800 m/z, and a scan time of 100 ms.

### Data processing

Mascot (Matrix Science, London, UK; version 2.3.02) was used for database searching of the MS/MS results and was set up to search SwissProt human database (20227 entries) assuming the use of trypsin. The parent and fragment ion mass tolerance was 0.05 Da. Carbamidomethyl cysteine was specified as a fixed modification, and 2 mis-cleavage sites were allowed. For protein identification, Scaffold (version Scaffold_4.3.2, Proteome Software Inc., Portland, OR) was used to validate MS/MS-based peptide and protein identifications. The protein identification was accepted at a false discovery rate (FDR) < 1.0% at the protein level and with at least 2 unique peptides. Proteins that contained similar peptides and could not be differentiated based on MS/MS analysis alone were grouped to satisfy the principles of parsimony. Scaffold Q+ (version Scaffold_4.3.2, Proteome Software Inc., Portland, OR) was used to perform the Label-Based Quantification (e.g., iTRAQ, TMT, SILAC) for peptide and protein identifications. The acquired intensities in the experiment were globally normalized across all runs. The reference channels were normalized to produce a 1:1-fold change, and all normalization calculations were performed using medians to normalize the data in a multiplicative fashion.

The technical CV of one sample for one protein was calculated as follows:

$${\mathrm{Technical CV}}_{\mathrm{sample}\;1}=\frac{\mathrm{standard deviation}\;\left(\mathrm{normalized intensity value of technical replication}\;1,\;\mathrm{technical variation}\;2,\;\mathrm{technical variation}\;3\right)}{\mathrm{average}\;\left(\mathrm{normalized intensity value of technical replication}\;1,\;\mathrm{technical variation}\;2,\;\mathrm{technical variation}\;3\right)}$$

The total technical CV was calculated by taking the average CV of each sample, as follows:

$$\mathrm{Technical CV}=\mathrm{mean}\;\left({CV}_{\mathrm{sample}\;1},\;{CV}_{\mathrm{sample}\;2},\ldots \right).$$

The inter-individual CV was calculated as follows:

$$\mathrm{Inter}-\mathrm{individual CV}=\frac{\mathrm{standard deviation}\;\left(\mathrm{mean normalized intensity value of individual}\;1,\;\mathrm{individual}\;2,..\right)}{\mathrm{average}\;\left(\mathrm{mean normalized intensity value of individual}\;1,\;\mathrm{individual}\;2,..\right)}$$

*Cluster* 3.0 software was used for hierarchical clustering analysis, and JAVA Treeview software was used to visualize clusters.

### Gene Ontology (GO) and Ingenuity Pathway Analysis (IPA) analysis

All proteins identified in the CSF and urinary samples were assigned a gene symbol using Panther database (http://www.pantherdb.org/). Protein classification was performed on the basis of functional annotations using GO for biological processes, molecular functions and cellular components.

The biomarker filter function in IPA software was used to filter the candidate CSF and urinary biomarkers. A disease and function analysis was used to examine the function of high-CV proteins in the urinary proteome.

## Results

### 1. Overall results of the urinary and CSF proteomes

In this study, 7 male samples and 7 female samples were digested by trypsin and labeled with 8-plex iTRAQ reagent; the pooled urinary/CSF samples were used as a control and analyzed by 2DLC-MS/MS for three times.

In the urinary proteome, a total of 749/721 protein groups were identified in the male/female urinary samples at FDR < 1% at protein level and with least 2 unique peptides (detailed data in Tables B and C in S1 File). A total of 968 protein groups were identified in urinary proteome. In the CSF proteome, a total of 436/419 protein groups were identified from the male/female CSF samples (detailed data are shown in Tables D and E in S1 File), and 512 protein groups were identified in all.

**Fig 2:**
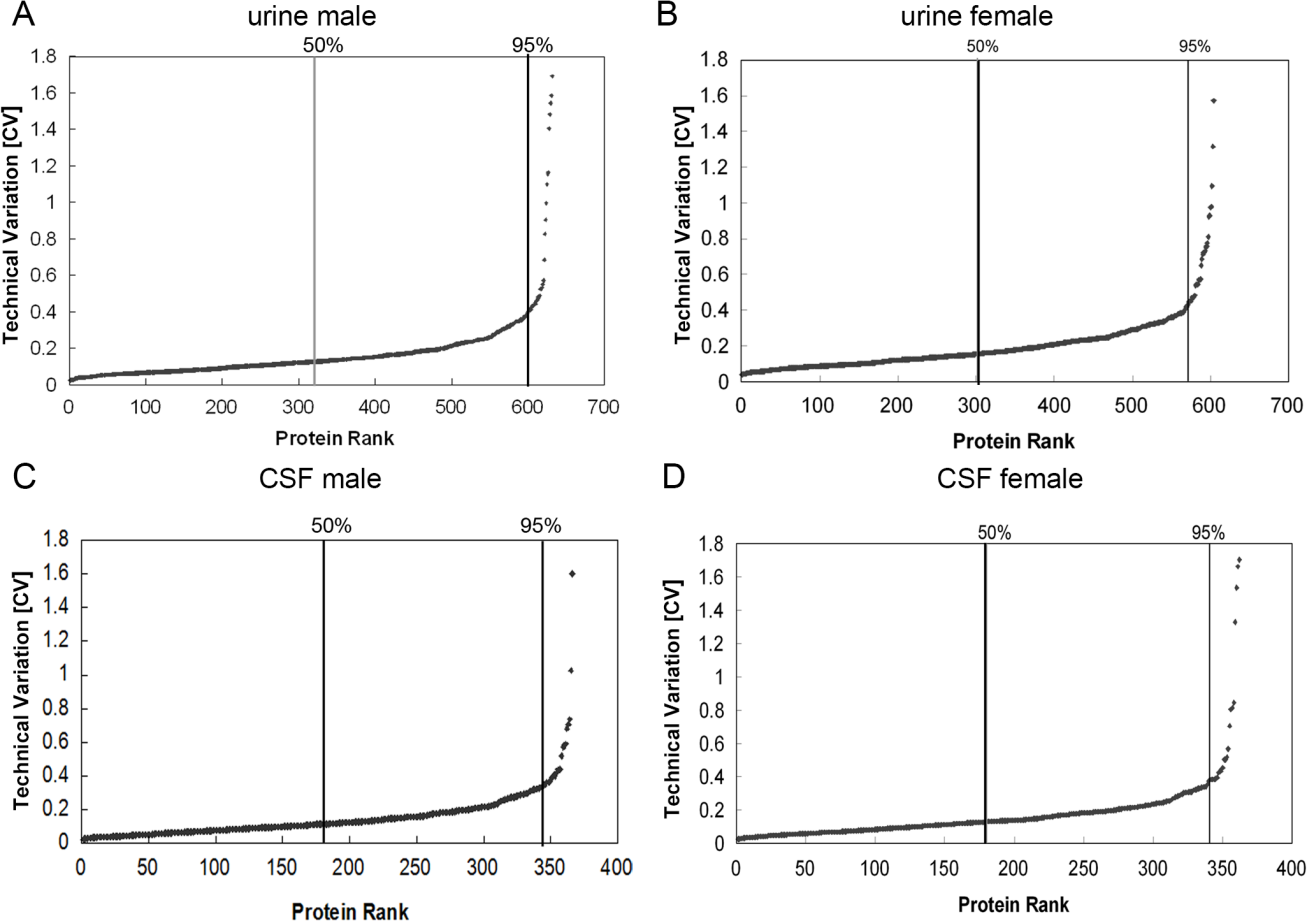
Technical variation in urinary and CSF proteomes. The technical variations in male (A) and female (B) urinary proteomes are shown. The technical variations in male (C) and female (D) CSF proteomes are shown. The proteins with median and top 5% technical CVs are annotated in the figures.

In the male/female urinary proteome, 632/605 proteins could be quantified in all 7 male/female samples in three replicates and were used for technical variation analysis (detailed data in Tables B and C in S1 File). Remarkably, the median CVs were 0.154/0.155 in the male/female urinary proteome, and 95% of the identifications (600/573) showed a technical CV lower than 0.406/0.455 (Fig 2A and 2B, Tables B and C in S1 File, Table 1). Technical variations in the CSF proteins were also estimated. In the male/female CSF samples, 366/362 proteins could be quantified in all 7 samples with three replicates (detailed data in Tables D and E in S1 File). The median technical CVs were 0.115/0.132, and 95% of the identifications (348/344) had technical CVs lower than 0.364/0.384, respectively, which were slightly lower than the CVs in the urinary proteome (Fig 2C and 2D, Tables D and E in S1 File, Table 1). To reduce the interference of technical variations, the proteins with the highest 5% technical CVs were excluded, and a total of 600/573 urinary proteins and 348/344 CSF proteins were used for an inter-individual variation analysis.

**Table 1:** The technical, inter-individual and intra-gender variations of the urinary and CSF proteomes.

	Technical CV	Inter-individual CV	Intra-gender CV
Male urine	15.40%	19.00%	26.20%
Female urine	15.50%	26.40%	
Male CSF	11.50%	15.90%	18.30%
Female CSF	13.20%	18.60%	

### 2. Individual variations in the urinary and CSF proteome

#### 2.1. Inter-individual variations

**Fig 3:**
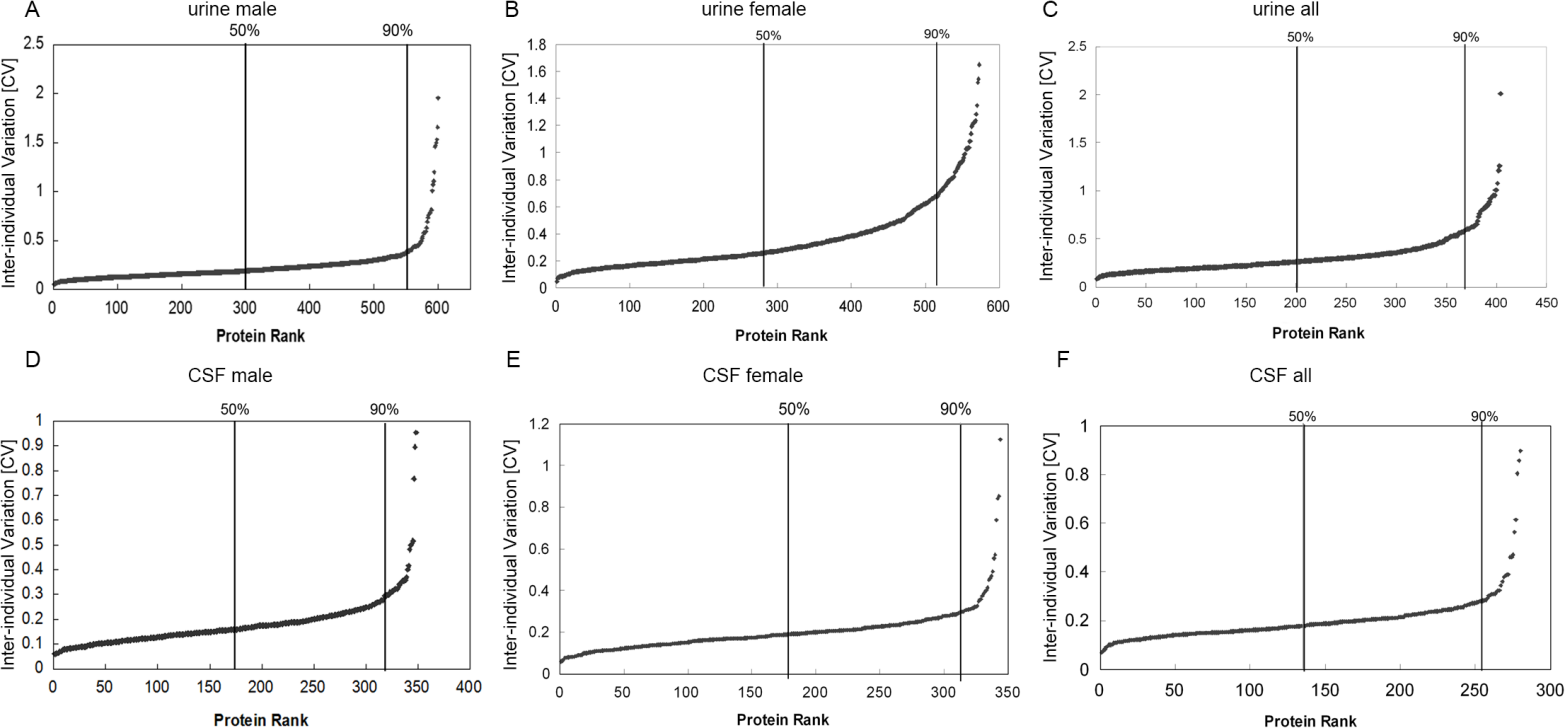
Inter-individual variation of urinary and CSF proteomes. The inter-individual variations of male (A), female (B), and both male and female (C) urinary proteomes are shown. The inter-individual variations of male (D), female (E), and both male and female (F) CSF proteomes are shown. The proteins with median and top 10% inter-individual CVs are annotated in the figures.

According to the analysis of 600/573 proteins in the urinary proteome, the medians of the inter-individual CVs were 0.190/0.264, and 90% had an individual CV lower than 0.347/0.677 (detailed data in Table F in S1 File). The inter-individual variation in the female urinary proteome was higher than that in the male urinary proteome (Fig 3A and 3B). In all, a total of 406 proteins were quantified in all 14 samples. Among the 406 proteins, 90% of the 363 proteins had an individual CV lower than 0.578, and the median individual CV was 0.262 (Fig 3C, Table F in S1 File, Table 1).

In the CSF proteome, the median inter-individual CVs from the analysis of 348/344 proteins were 0.159/0.186, and 90% of the CSF male/female proteins had individual CVs lower than 0.271/0.286, lower than those of the urinary proteome (Fig 3D and 3E, detailed data in Table F in S1 File). Among the 512 identified proteins, a total of 280 could be quantified in all 14 CSF samples. Ninety percent (253 proteins) showed inter-individual values lower than 0.276, and the median inter-individual result was 0.183 (Fig 3F, Table F in S1 File, Table 1).

#### 2.2. Correlation of technical and individual variations

The technical and individual variations were plotted against each other: spots lying close to the 45° line are those proteins that have an inter-individual CV similar to that of the technical CV. As shown in S1A and S1B Fig, the great majority of proteins in both the male and female urinary proteomes had an inter-individual CV that was larger than the technical CV (71.4%/74.5% of male/female proteins). The CSF proteome presented similar results: 69.3%/70.8% of male/female CSF proteins showed inter-individual CVs larger than the technical CV (S1C and S1D Fig). These results indicated that the technical variation made only a partial contribution to the total variation, as measured in different individuals. S1 Fig also indicates that there were no positive correlations between the technical variation and the inter-individual variation.

#### 2.3. Correlating individual variation and protein abundance

Spectral counts were used to evaluate protein abundance in this study. The Log_2_ transformed spectral count for each protein was plotted against the corresponding inter-individual variation. No correlations between inter-individual CVs and protein abundance in the male and female urinary samples were found (S2A and S2B Fig). Some highly abundant proteins (spectral counts >32) also showed high inter-individual variations (CV>0.5), such as alpha-2-HS-glycoprotein in the female urinary proteome and kallikrein-1 in the male proteome. Approximately 95.7%/84.6% of low-abundance proteins (spectral counts <5) in the male/female urinary proteome showed low inter-individual variations (CV<0.5). Similar to the results from the urinary proteome, the individual protein variations did not correlate with abundance in the male and female CSF proteomes (S2C and S2D Fig). These findings suggested that the inter-individual variations were not correlated with protein abundance.

#### 2.4. Comparison of the individual variation of the urinary and CSF proteomes

**Fig 4:**
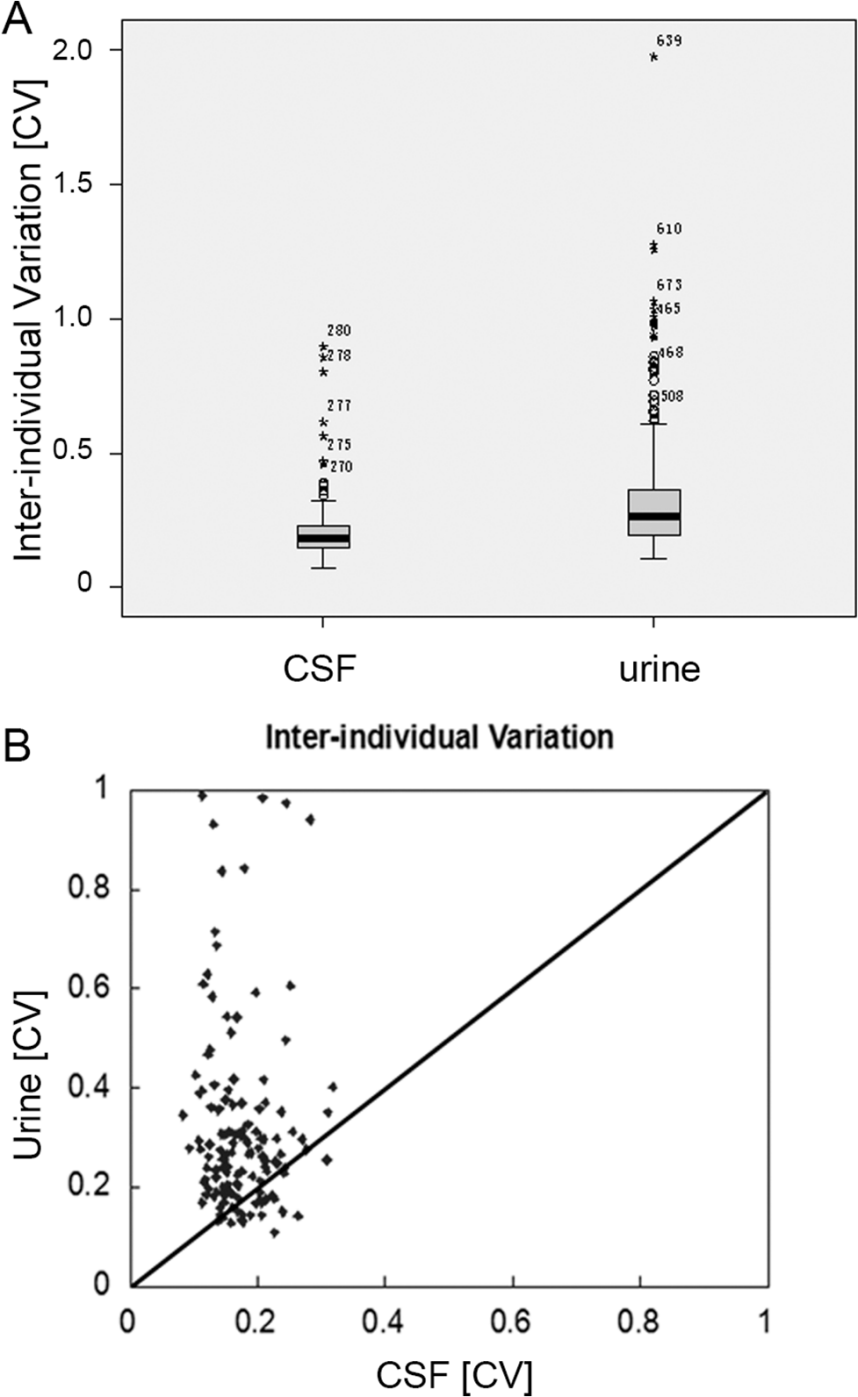
Comparison of the inter-individual variation of urinary and CSF proteomes. A. Distribution of inter-individual variations of CSF and urine are shown in a box plot. The inter-individual variation of CSF was much lower than that of urine. B. Scatter plots of the inter-individual variation for urine and CSF proteins. The CV determined for the inter-individual variation of CSF is plotted against the inter-individual variation of urine. The spots above the 45° line show a higher CV for urine than CSF, which is true for the vast majority of the spots.

A total of 406 urinary proteins and 280 CSF proteins were quantified in 14 urinary and CSF samples and then used for individual variation comparisons. Fig 4A shows that the inter-individual variation of urinary proteins (median CV = 0.262) was significantly higher than that of CSF proteins (median CV = 0.183) (p<0.05). A total of 139 proteins could be quantified in the 14 CSF and 14 urinary samples. The inter-individual CVs of 139 CSF and urinary proteins were plotted against each other. As shown in Fig 4B, approximately 80% of the proteins had lower inter-individual variations in CSF compared to urine. These results indicated that CSF proteome was more stable than urinary proteome.

### Inter-gender variation

**Fig 5:**
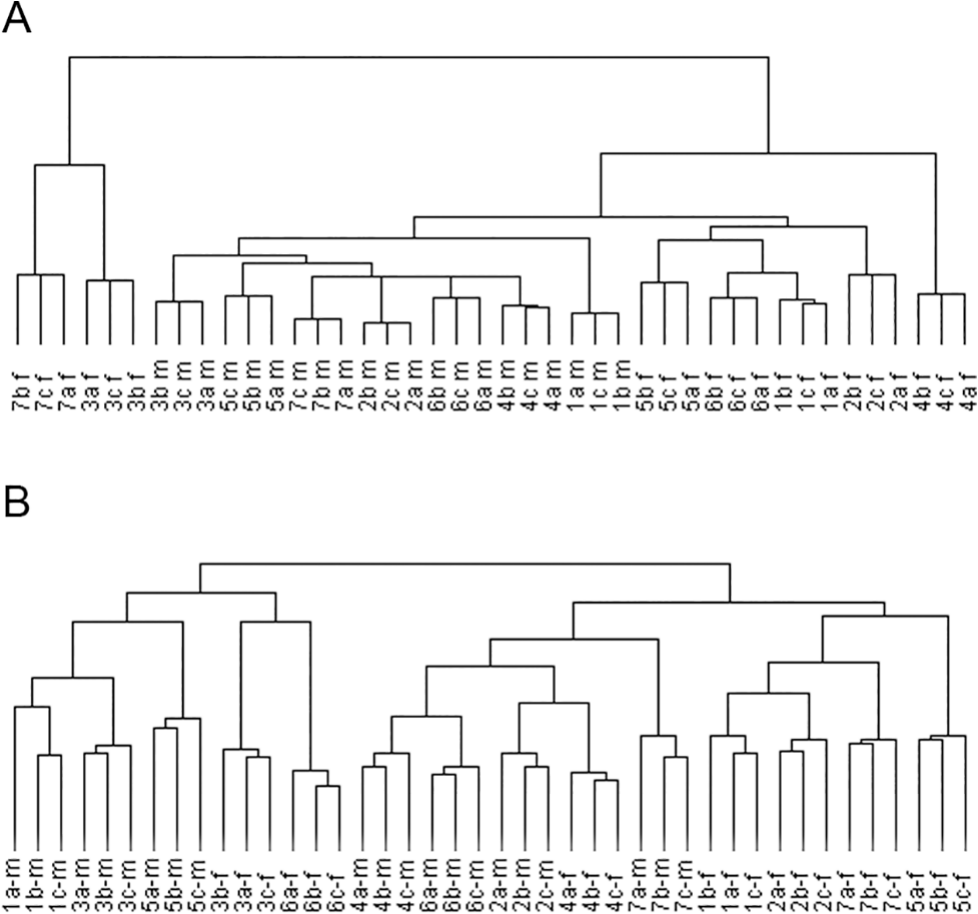
Inter-gender variation in the urinary and CSF proteomes. A. Unsupervised hierarchical clustering of triplicate samples from 14 cognitively normal individuals (7 males and 7 females). B. Unsupervised hierarchical clustering of triplicate samples from 14 cognitively normal individuals (7 males and 7 females).

A hierarchical clustering analysis was performed to comprehensively define inter-individual and inter-gender variation in urinary proteome. As shown in Fig 5A and S3A Fig, all technical replicates within one sample were clustered together, demonstrating that technical variation was less than inter-individual variation. Moreover, male samples could be clustered into one group. These results indicated that male and female urinary proteomes exhibited different patterns. The above result was similar to that in our previous study [1].

To define the proteins causing inter-gender variations, proteins that were differentially expressed between the male and female urinary proteomes were studied. Between the male and female samples, a total of 20 proteins showed more than a 2-fold change and had a p-value <0.05 (Table G in S1 File). The highly expressed male proteins were male-specific proteins of the urinary system, such as prostate-origin proteins, beta-microseminoprotein, prostate-specific antigen and prostatic acid phosphatase. The highly expressed female proteins were associated with lipid and carbohydrate metabolism-related proteins, such as carbonic anhydrase 1, APOA4, APOC3, and lipocalin-1.

A hierarchical clustering analysis was also performed for CSF proteome. Unlike in urinary proteome, the male and female CSF samples could not be distinguished by clustering (Fig 5B and S3B Fig). These results indicated that there were no distinct differences between the male and female samples.

### 4. Minimum sample sizes for differential proteomics

The minimum urinary sample sizes were estimated using the method described in our previous study [1]. The median inter-individual variations for quantitative proteins were 0.262 and 0.183 in urine and CSF, respectively. The minimum sample size at 5% FDR is shown in Table 2. When the fold change was 2, the average minimum sample size was approximately 5 for the urinary samples and 4 for the CSF samples, as based on iTRAQ labeling and 2DLC/MS/MS analysis. Because the patterns of the urinary proteome were different between males and females, we estimated the minimum sample size for the male and female urinary samples separately and obtained average minimum sample sizes of 4 and 5 for male and female urinary samples (Table H in S1 File).

**Table 2:** The estimated minimal sample size for quantitative analysis based on the iTRAQ labeling quantification method for urine (A) and CSF (B), where power is the power of the statistical test, α is the significance level, and π refers to the estimated proportion of truly deferentially expressed proteins among all of the identified proteins.

A					
fold change	power	a	π	FDR	**n**
2	0.8	0.001	0.05	2.32%	6
2	0.8	0.005	0.1	5.33%	5
2	0.8	0.01	0.2	4.76%	5
2	0.9	0.001	0.05	2.06%	6
2	0.9	0.005	0.1	4.76%	5
2	0.9	0.01	0.2	4.26%	5
B					
fold change	power	a	π	FDR	**n**
2	0.8	0.001	0.05	2.32%	4
2	0.8	0.005	0.1	5.33%	4
2	0.8	0.01	0.2	4.76%	3
2	0.9	0.001	0.05	2.06%	5
2	0.9	0.005	0.1	4.76%	4
2	0.9	0.01	0.2	4.26%	4

## Discussion

### Correlation of individual protein variations and functions

**Fig 6:**
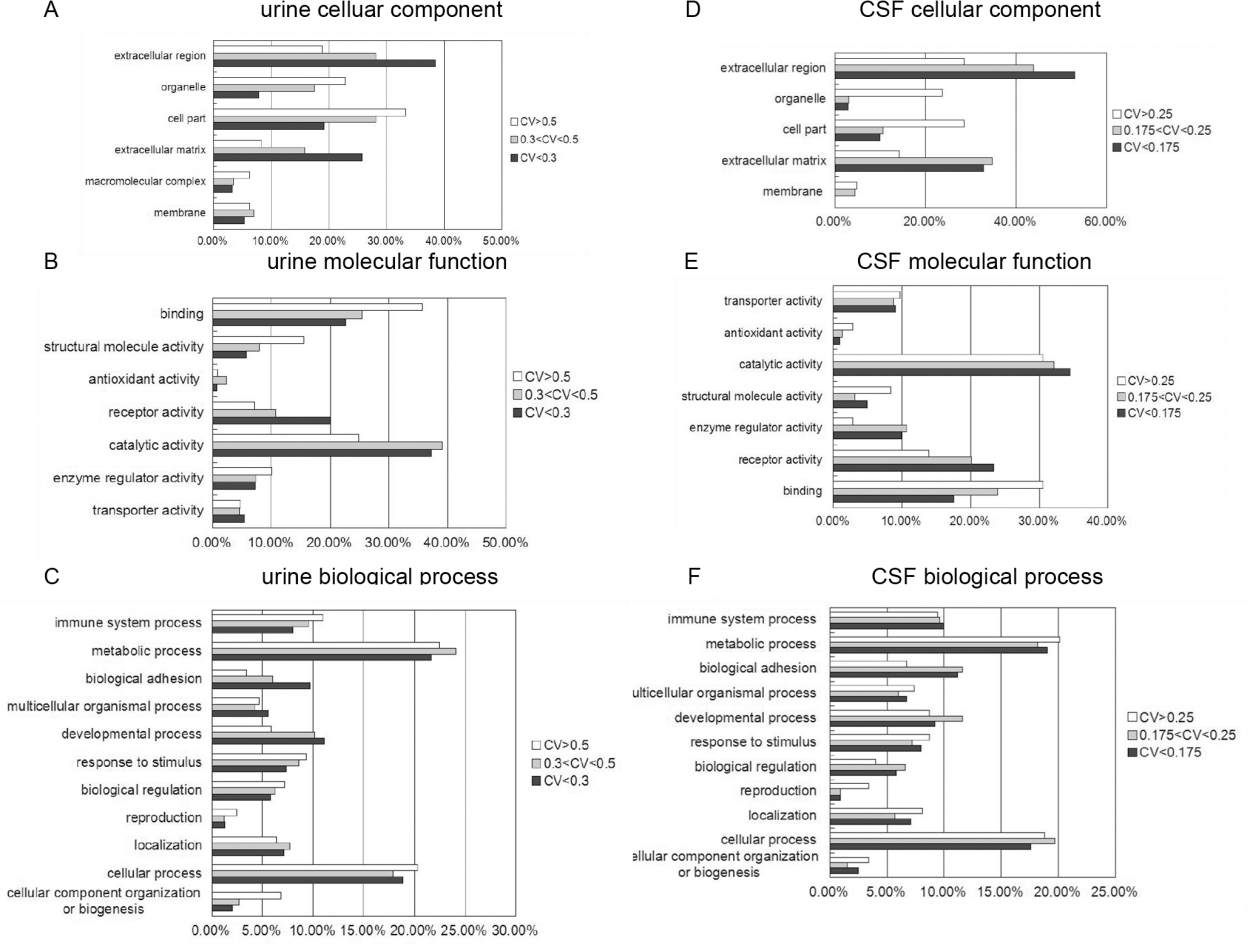
GO term enrichment analysis of high-CV proteins and low-CV proteins in the urinary and CSF proteomes. High, mid and low individual CV urinary proteins are classified into cellular component (A), molecular function (B) and biological process (C) categories for human genes. Genes for which no annotations could be assigned were excluded from the analysis for both the ligand and the genome sets. Categories with a constitution of at least 2% are displayed in the bar charts. High, mid and low individual CV CSF proteins are classified into cellular component (D), molecular function (E) and biological process (F) categories for human genes.

In this study, we compared inter-individual and inter-gender variations in urinary and CSF proteomes based on iTRAQ quantification analysis. In the urinary proteome, a number of proteins were found to be stable among different individuals. Indeed, 60.5% of the quantified proteins (246 of 406) had an inter-individual variation of less than 0.3, and 13.3% of the quantified proteins (54 of 406) showed high variations (>0.5). GO analysis showed that extracellular proteins, such as receptor proteins and biological adhesion-related proteins, tended to have lower CVs, whereas intracellular proteins, such as organelle proteins, macromolecular proteins and structure proteins, tended to have higher CVs (Fig 6A–6C). The extracellular proteins were primarily secreted from the urinary tract or originated from plasma; thus, they exhibited relatively good stability. The intracellular proteins identified were released from exfoliative cells originating from aged or damaged tissue in the kidneys, ureter, bladder, and the urethra of the urinary system [17], and these proteins were relatively unstable. We further studied the stability of secreted enzymes and found that 35 of the 425 urinary proteins are secreted enzymes. The average CV of these enzymes was 0.346, which is similar to the average CV of the non-enzyme proteins (0.331). These results indicate that many secreted enzymes could stably exist in urine. Havanapan et al.[18] observed neither quantitative nor qualitative changes were observed in urinary samples that were stored without protease inhibitors, suggesting that these proteases might be deactivated in urine; however, more robust evidence should be presented in the future work. According to IPA analysis, many high-CV proteins were related to lipid metabolism, including apolipoprotein A-I (0.93) and apolipoprotein A-IV (0.54) (Table I in S1 File). As these proteins could be affected by diet and lifestyle, they differ among individuals by a large degree. There were also high variations in gender-related proteins, such as prostate-specific antigen (0.562) and uteroglobin (1.064).

In the CSF proteome, most proteins were stable among the individuals. Additionally, 92.1% of the quantified proteins (258 of 280) showed an inter-individual variation of less than 0.3. According to GO analysis, similar to the urinary proteome, extracellular proteins originating from plasma or secreted from the CNS tended to have lower CVs, whereas intercellular proteins, released from exfoliative cells in the CNS, tended to have higher CVs (Fig 6D–6F). Specifically, many high-abundance proteins from blood showed low inter-individual variation, such as albumin (CV = 0.113), serotransferrin (0.132) and ceruloplasmin (0.160). These proteins have roles in homeostasis in the CNS. The majority of CNS-specific proteins showed moderate inter-individual CVs, such as neuroserpin (0.24), neuronal growth regulator 1 (0.225), and neuronal cell adhesion molecule (0.200). These proteins are secreted from the CNS and vary among different individuals, but they are present within a certain range in healthy people. According to IPA analysis, the proteins related to the inflammatory response showed the largest inter-individual CVs. These proteins include acute-phase response signaling-related proteins, such as haptoglobin (0.899); alpha-1-acid glycoprotein 1 and 2 (0.563 and 0.461); fibrinogen alpha (0.389), beta (0.470) and gamma (0.388) (Table I in S1 File); and immunoglobulins, such as WFIKKN2 (0.389) and IGHA1 (0.615). By analyzing the quantitative distribution between inflammatory disorder group (bone fracture, urinary calculus and acute appendicitis) and non-inflammatory disorder group (varicose, fibroid and oophoritic cysts), we did not find significant differences with regard to these 8 proteins. The high CV of these proteins was caused by a few individuals with remarkably high abundance but not by inflammation.

### Inter-individual variations in candidate biomarkers

A total of 142 urinary proteins identified in this study were identified as candidate biomarkers by IPA analysis. The median inter-individual CV of these proteins was 0.3, and 20 of these proteins had a CV higher than 0.6. Some candidate biomarkers for kidney disease also exhibited high variations, such as beta-2-microglobulin (1.26), zinc alpha-2-glycoprotein (0.98) and alpha-2-HS-glycoprotein (0.84). A total of 26 CSF proteins were identified as candidate biomarkers for neurological or psychological diseases or CNS tumors by IPA analysis, and the median inter-individual CV of these proteins was 0.177; only TTR and CHI3L1 had CVs slightly higher than 0.3 (Table J in S1 File).

The above results showed that the CVs of potential biomarkers were similar to that of the overall proteome. Proteins with high CVs could also be used as biomarkers, but more samples and higher thresholds should be used.

### Comparison of different quantification methods for inter-individual variation

Several studies have employed 1DLC/MS/MS and label-free methods for individual variation analysis; therefore, we compared our results with previous results (Table 3). For the urinary proteome, the spectral count method [1] showed the highest inter-individual CV and minimum sample size, whereas the iTRAQ quantification method showed the lowest; the results from the peak intensity label-free method [11] were between these values. Regarding the CSF proteome, the peak intensity-based label-free method was performed in two different laboratories [13,15], and the results of both showed higher inter-individual CVs and higher minimum sample sizes compared to the iTRAQ quantification method.

**Table 3:** Comparison of the inter-individual variations and minimal sample sizes of the urinary and CSF proteomes between this study and previous studies.

		Quantification method	Inter-individual variation	Minimal sample size (2-fold)
Urine	Nagaraj et al.[11]	Label-free	66%	16
	Liu et al.[1]	Spectrum counting	71%	18
	Our study	iTRAQ	26.20%	5
CSF	Stoop et al.[13]	Label-free	42%	8
	Perrin et al.[15]	Label-free	55%	12
	Our study	iTRAQ	18.30%	4

The label-free method is based on peak intensity and is quantified using MS1 signal. However, MS1 data are less accurate and specific, and impurity peaks with the same m/z values might be falsely calculated into the target peptide; therefore, this method might exaggerate the true variations [19,20]. The iTRAQ quantification method is quantified using MS2, and such quantifications provide higher accuracy and specificity, though impurity interference would compress true variations [21,22]. As true inter-individual variations might fall between the value from the label-free method and that from the iTRAQ method, the minimum sample size with a 2-fold change for proteomic analysis might be 10/8 for the urinary/CSF proteome.

### Inter-gender variation comparison of urine and CSF proteomes

According to our analysis, the male and female samples could be separated by hierarchical clustering analysis, indicating significant differences between the male and female urinary proteome patterns. The above result was supported by our previous study employing 1DLC/MS/MS analysis and the spectral count method [1,11]. The males highly expressed prostate-originating proteins, and the females highly expressed some lipid and carbohydrate metabolism-related proteins, which could partially explain the difference between the male and female urinary proteomes. Therefore, it is important that the ratio of male to female samples be balanced for differential proteomic analysis. In contrast, there were no remarkable differences in the CSF proteome patterns between males and females.

Interestingly, we found that the female urinary proteome has larger inter-individual variations than the male proteome. By using IPA analysis, we found that the high variation of proteins in females was associated with cell movement, cell migration and inflammatory responses (Table I in S1 File). However, identification of the reason for these differences requires further work.

### Inter-individual variations between the CSF and urine proteomes

With regard to both the overall inter-individual CV and individual CV of a certain protein, that of the urinary proteome was higher than that of the CSF proteome. Urine is a liquid product that is secreted by the kidneys and excreted through the urethra. Urine is used for waste excretion and body homeostasis regulation. Therefore, the urinary proteome could be affected by exercise, diet, lifestyle and other factors and may exhibit relatively large variations. In contrast, CSF provides a chemically stable environment for the CNS; therefore, the stability of the CSF is necessary, and the CSF proteome shows low variation.

In the clinic, CSF is not easily accessible, and plasma is too complex for proteomic analyses. In this study, some proteins could be co-identified in both the urinary and CSF proteomes; thus, urine could be an alternative choice for CNS disease biomarker discovery. In 2012, Rodríguez-Rodríguez et al. [23] found that S100B in urine could be used as a mortality predictor after severe traumatic brain injury. In this study, 14 candidate biomarkers for CNS diseases were quantified in both CSF and urine (Table J in S1 File). These proteins have the potential to serve as CNS disease biomarkers in urine instead of CSF.

## Conclusions

In this study, 2DLC-MS/MS analysis and iTRAQ quantification were used to evaluate inter-individual and inter-gender variations as well as the minimum sample size of the urinary and CSF proteomes. A statistical analysis showed that the urinary proteome exhibited gender differences, whereas the CSF proteome did not. The urinary proteome also presented higher individual variations than did the CSF proteome. In both urinary and CSF proteomes, intra-cellular proteins tended to have high CVs, whereas extracellular proteins tended to have low CVs.

Therefore, in urinary proteome studies, the ratio of male to female samples should be balanced for differential proteomic analysis. The average minimum sample size was 10 with 2-fold change. In CSF proteome study, the balance between males and females was not very important, but examining both male and female samples was necessary; the average minimum sample size was 8 with a 2-fold change. The results of this work will benefit the design of body fluid proteomic analyses.

## Supporting Information

S1 Fig The correlation of technical and individual variation. Scatter plots of technical and inter-individual variations for male (A) and female (B) urinary proteins. Scatter plots of technical and inter-individual variations for male (C) and female (D) CSF proteins. The CV determined for the inter-individual variation is plotted against the CV for technical variation. Spots below the 45° line show higher inter-individual variance than technical variance, which is true for the vast majority of all spots.
(TIF)

S2 Fig The correlation of protein abundance and individual variation. Plots of inter-individual CV against the abundance of proteins (Log2-transformed spectral counts) for male (A) and female (B) urinary proteins and male (C) and female (D) CSF proteins are shown.
(TIF)

S3 Fig Inter-gender variation in the urinary and CSF proteomes (in detail). A. Unsupervised hierarchical clustering of triplicate samples from 14 cognitively normal individuals (7 males and 7 females) and 404 urine proteins. B. Unsupervised hierarchical clustering of triplicate samples from 14 cognitively normal individuals (7 males and 7 females) and 280 CSF proteins. red = high, black = mean value, green = low.
(PDF)

S1 File Supplementary Tables. Table A, The ages of each of the 7 male and 7 female participants who provided urine samples and CSF samples. Table B, Protein and peptide quantification data and technical variation data for male urine. The Log2 transformed iTRAQ quantification values for every sample are shown in the “Quantitative protein urine male” sheet, and the technical CVs for the quantitative proteins are shown in the “Quantitative protein urine male” sheet. The peptide relative quantification values are shown in the “Quantitative peptide urine male” sheet. Table C, Protein and peptide quantification data and technical variation data for female urine. The Log2 formed iTRAQ quantification values for every sample are shown in the “Quantitative protein urine F” sheet, and the technical CVs for the quantitative proteins are shown in the “Quantitative protein urine F” sheet. The peptide relative quantification values are shown in the “Quantitative peptide urine F” sheet. Table D, Protein and peptide quantification data and technical variation data for male CSF. The Log2 formed iTRAQ quantification values for every sample are shown in the “Quantitative protein male CSF” sheet, and the technical CVs for the quantitative proteins are shown in the “Quantitative protein male CSF” sheet. The peptide relative quantification values are shown in the “Quantitative peptide male CSF” sheet. Table E, Protein and peptide quantification data and technical variation data for female CSF. The Log2 formed iTRAQ quantification values for every sample are shown in the “Quantitative protein female CSF” sheet, and the technical CVs for the quantitative proteins are shown in the “Quantitative protein female CSF” sheet. The peptide relative quantification values are shown in the “Quantitative peptide female CSF” sheet. Table F, Inter-individual variation data for urinary and CSF samples. The inter-individual CV and technical CV for male urine, female urine, urine (both male and female), male CSF, female CSF, CSF (both male and female) and both CSF and urine are shown in the respective sheets. Table G, Proteins that were differentially expressed between the female and male urinary proteomes. The p-value and the ratio (female/male) for the differentially expressed proteins are shown. Table H, The estimated minimal sample size for quantitative analysis based on the iTRAQ labeling quantification method for urine in males and females, where power is the power of the statistical test, α is the significance level, and π refers to the estimated proportion of truly differentially expressed proteins among all of the identified proteins. Table I, A functional analysis of high-CV proteins in the urinary proteome, female urinary proteome and CSF proteome by IPA is shown in the “urine high CV” sheet, “female urine high CV” sheet and “CSF high CV” sheet, respectively. The disease and function terms, p-value, and related molecules are shown. The acute-phase response signaling-related high-CV CSF proteins are shown in the “CSF high CV” sheet. Table J, Inter-individual variation data for biomarkers from CSF and urine, as filtered by IPA analysis. The candidate biomarkers in urine and CSF and the candidate CNS disease biomarkers in CSF filtered by IPA are shown in the “urine”, “CSF” and “CSF CNS-disease” sheets, respectively. The inter-individual CVs of these proteins are shown. The candidate CNS disease biomarkers identified in both CSF and urine are shown in the “CSF-urine-CNS disease” sheet.
(ZIP)
