# Correction: A Proteomic Analysis of Individual and Gender Variations in Normal Human Urine and Cerebrospinal Fluid Using iTRAQ Quantification

**DOI:** 10.1371/journal.pone.0213213

**Published:** 2019-04-01

**Authors:** Zhengguang Guo, Yang Zhang, Lili Zou, Danqi Wang, Chen Shao, Yajie Wang, Wei Sun, Liwei Zhang

Following publication of [1] concerns were raised that the urine proteomics data reported do not correlate to other findings reported in the literature. Specifically:

- Semenogelin was reported to be abundantly found in female urine.
- Albumin and Uromodulin are not detected as the most abundant proteins in urine.

The authors note that Semenogelin-1 could be identified in both male and female samples, though female urine showed about 20 times lower level than male urine. The mean relative abundance of Semenogelin-1 in female urine is 0.23, and the mean relative abundance of Semenogelin-1 in male urine is as high as 4.05; note that all values are relative to the pooled control containing a mixture of both male and female urine, as described below.

To further clarify the results, the authors wish to add the following additional information about sampling within the Methods section:

“All urine samples (7 males and 7 females) were used as one pooled sample and all CSF samples (7 males and 7 females) were used as another pooled sample. In one pool, the mixed urine sample (including 7 males and 7 females) was labeled with 113. The 7 male samples were individually labelled with 114, 115, 116, 117, 118, 119 and 121 iTRAQ reagents. In another pool, the mixed urine sample (including 7 males and 7 females) was labeled with 113, and the 7 female samples were individually labelled with 114, 115, 116, 117, 118, 119 and 121 iTRAQ reagents. The individual male and female urines were analyzed separately, but the mix pooled urine sample contains both male and female urine samples.”

Most female samples show missing data or low Log2 ratios as calculated from comparison with the pooled sample, but the protein levels are reported in values that are significantly higher than the peptide results. In the Supplementary Tables B-E of S1 File from [1], the peptide levels are presented in the Log2 ratios compared to the mix pooled sample, but the protein levels are presented as peak intensity values. Therefore, the protein results seem to show significantly higher values than those of the peptide. When they are both presented as Log2 ratios, the quantification result of the peptide and protein show similar trends (S1–S4 Data).

The authors wish to clarify that 95% peptide probability as a cut-off was more stringent and did not allow a comprehensive report of all peptides. Instead, the authors used the protein false discovery rate algorithm, which can reliably estimate false discovery rate (FDR) for protein identification in large scale datasets [2] and provide a consistent assessment of the data generated from different software. This was also recommended by the guideline for credible proteomic identification [3, 4]. The filter criteria the authors used is as follows: the protein identification is accepted with FDR < 1.0% at both the peptide and the protein level, and each protein with at least 2 unique peptides [3, 4].

To compare the data reproducibility across different platforms, especially the most abundant components like albumin and uromodulin, firstly, the authors wish to clarify that they compared the qualitative urinary proteome results between their dataset and other datasets [1, 5, 6]. 98% of the identified proteins in the authors’ datasets overlap with other datasets (S4A Fig). Next, the authors compared the numbers of normalized spectral counts in their study with other publications across platforms [1, 5]. In spectral counting levels, albumin and uromodulin are the highest abundance proteins in the authors’ data, and the normalized spectral counts of the highest abundance proteins are similar between their study and Zheng’s study [1, 5] (S4B Fig). The difference between the authors’ study and other publications might be caused by differences of the sample preparation, quantification method, MS platform, and the individual variations of the study participants.

The authors wish to provide additional methodological details about how protein concentrations were determined in [1]:

“The protein concentration was determined by Bradford method. For the preparation of a standard curve: 1 μg/uL BSA solution was prepared in lysis buffer (7 M urea, 2 M thiourea, 0.1 M DTT, and 50 mM Tris), and diluted into 0.1, 0.2, 0.3, 0.4 and 0.5 μg/μL.

Urine samples were precipitated overnight at -4 °C after addition of 3 volumes of ethanol. After 30 minutes of centrifugation at 10,000 x g, the pellets were re-suspended in lysis buffer. 10 μL (0.1, 0.2, 0.3, 0.4 and 0.5 μg/μL) BSA and the test sample were added into a 96 well microplate, respectively. The 10 μL lysis buffer was added into a 96 well microplate as blank. 10 μL urine protein sample was also added into a 96 well microplate. Each sample and standard curve was prepared in three parallel wells. 200 μl Bradford working fluid were added in each well containing sample and standard, slow shaking in room temperature. The OD value in 595 nm of each well was read in Epoch Microplate reader (Biotek, USA). We used the OD value in 595 nm of the BSA standard to plot the standard curve. The R^2^ of the standard curve should be 0.95–0.99. Using the formula of the standard curve and the OD value of the test sample, the protein concentration of the test sample was calculated.”

The proteomics data have been uploaded to http://www.iprox.org. (Project Name: A proteomic analysis of individual and gender variations in normal human urine and cerebrospinal fluid using iTRAQ quantification, Project ID: IPX0001396000, http://www.iprox.org//page/SCV017.html?query=IPX0001396000).

## Supporting information

S1 Data Protein quantification data for male urine. (XLS)

S2 Data Protein quantification data for female urine. (XLS)

S3 Data Protein quantification data for male CSF. (XLS)

S4 Data Protein quantification data for female CSF. (XLS)

S4 Fig A: Venn diagram of the identified proteins in our dataset and other publications. B: Comparison of the normalized spectral counts for most abundant proteins and in our dataset and in other publication.
(DOCX)
