# Supplementary figures and images for: A Proteomic Analysis of Individual and Gender Variations in Normal Human Urine and Cerebrospinal Fluid Using iTRAQ Quantification
Source: PLoS One. 2015 Jul 29;10(7):e0133270. doi: 10.1371/journal.pone.0133270 (PMC4519152; doi:10.1371/journal.pone.0133270)

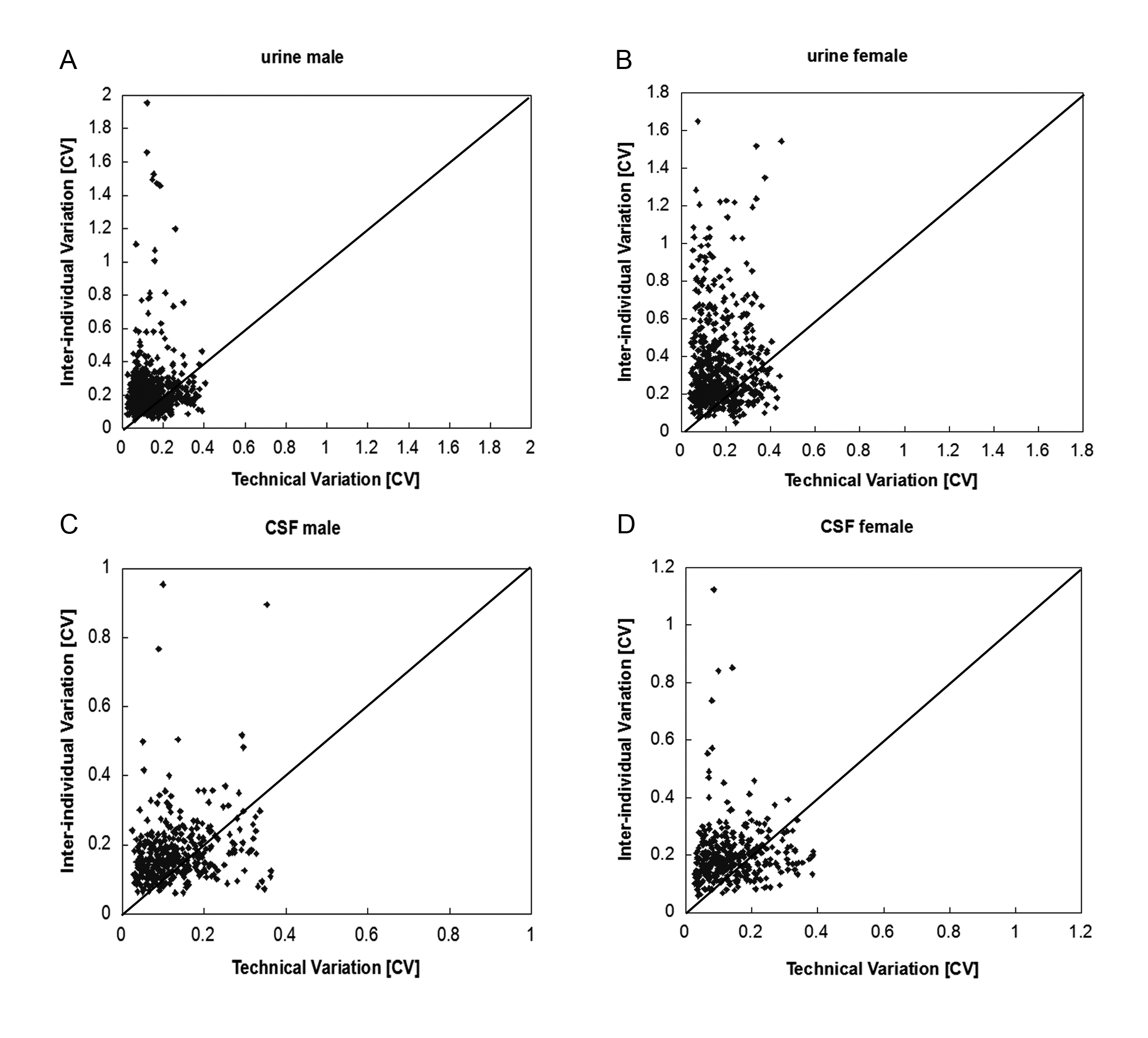

Supplement: S1 Fig — Scatter plots of technical and inter-individual variations for male (A) and female (B) urinary proteins. Scatter plots of technical and inter-individual variations for male (C) and female (D) CSF proteins. The CV determined for the inter-individual variation is plotted against the CV for technical variation. Spots below the 45° line show higher inter-individual variance than technical variance, which is true for the vast majority of all spots. (TIF) [file pone.0133270.s001.tif]

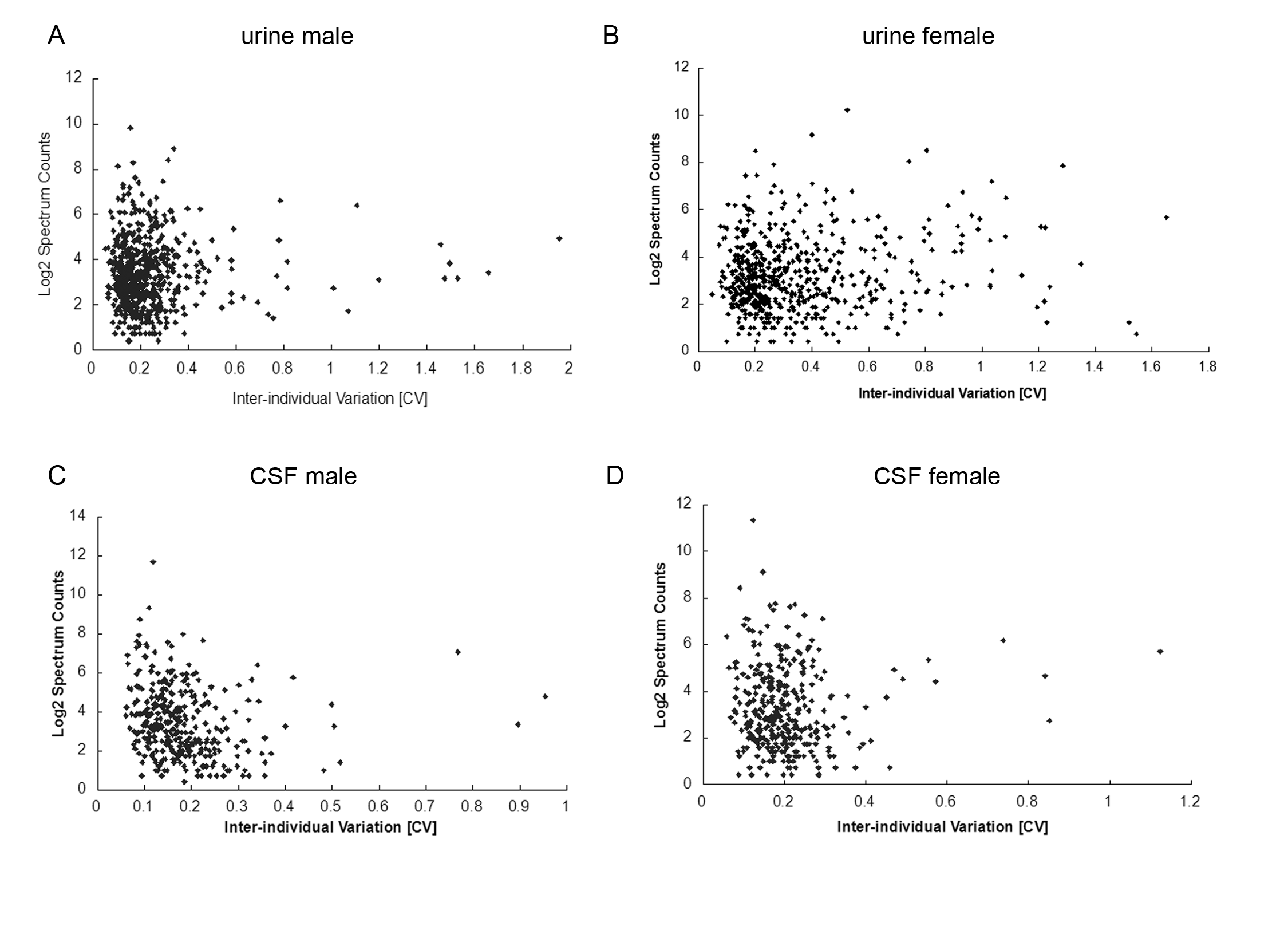

Supplement: S2 Fig — Plots of inter-individual CV against the abundance of proteins (Log2-transformed spectral counts) for male (A) and female (B) urinary proteins and male (C) and female (D) CSF proteins are shown. (TIF) [file pone.0133270.s002.tif]

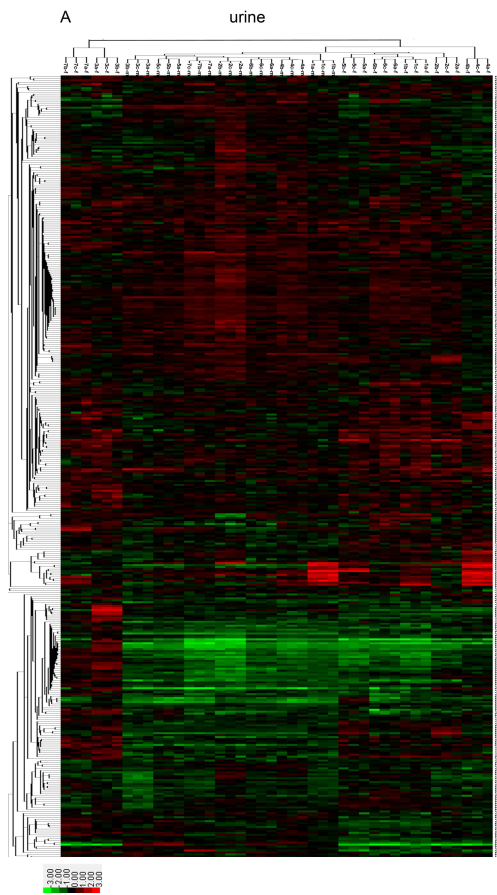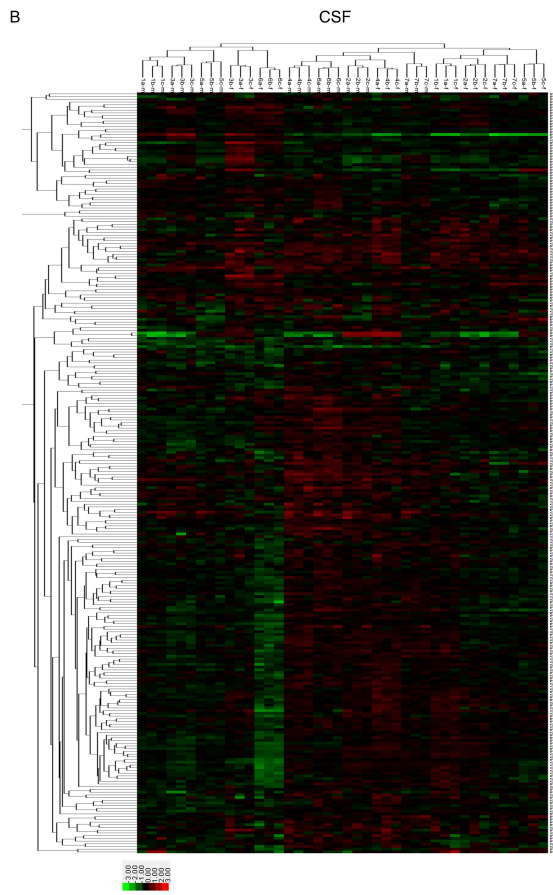

Supplement: S3 Fig — A. Unsupervised hierarchical clustering of triplicate samples from 14 cognitively normal individuals (7 males and 7 females) and 404 urine proteins. B. Unsupervised hierarchical clustering of triplicate samples from 14 cognitively normal individuals (7 males and 7 females) and 280 CSF proteins. red = high, black = mean value, green = low. (PDF) [file pone.0133270.s003.pdf]
