# Supplementary material for: A Proteomic Analysis of Individual and Gender Variations in Normal Human Urine and Cerebrospinal Fluid Using iTRAQ Quantification
Source: PLoS One. 2015 Jul 29;10(7):e0133270. doi: 10.1371/journal.pone.0133270 (PMC4519152; doi:10.1371/journal.pone.0133270)
Supplement: S1 File — Table A, The ages of each of the 7 male and 7 female participants who provided urine samples and CSF samples. Table B, Protein and peptide quantification data and technical variation data for male urine. The Log2 transformed iTRAQ quantification values for every sample are shown in the “Quantitative protein urine male” sheet, and the technical CVs for the quantitative proteins are shown in the “Quantitative protein urine male” sheet. The peptide relative quantification values are shown in the “Quantitative peptide urine male” sheet. Table C, Protein and peptide quantification data and technical variation data for female urine. The Log2 formed iTRAQ quantification values for every sample are shown in the “Quantitative protein urine F” sheet, and the technical CVs for the quantitative proteins are shown in the “Quantitative protein urine F” sheet. The peptide relative quantification values are shown in the “Quantitative peptide urine F” sheet. Table D, Protein and peptide quantification data and technical variation data for male CSF. The Log2 formed iTRAQ quantification values for every sample are shown in the “Quantitative protein male CSF” sheet, and the technical CVs for the quantitative proteins are shown in the “Quantitative protein male CSF” sheet. The peptide relative quantification values are shown in the “Quantitative peptide male CSF” sheet. Table E, Protein and peptide quantification data and technical variation data for female CSF. The Log2 formed iTRAQ quantification values for every sample are shown in the “Quantitative protein female CSF” sheet, and the technical CVs for the quantitative proteins are shown in the “Quantitative protein female CSF” sheet. The peptide relative quantification values are shown in the “Quantitative peptide female CSF” sheet. Table F, Inter-individual variation data for urinary and CSF samples. The inter-individual CV and technical CV for male urine, female urine, urine (both male and female), male CSF, female CSF, CSF (both m [file pone.0133270.s004.zip › Supplementary Table/Table A.doc]

Table A The ages of each of the 7 male and 7 female participants who provide urine samples and CSF samples.

Urine Samples

| ID | age |
| --- | --- |
| m1 | 28 |
| m2 | 27 |
| m3 | 30 |
| m4 | 28 |
| m5 | 26 |
| m6 | 23 |
| m7 | 35 |
| f1 | 26 |
| f2 | 26 |
| f3 | 23 |
| f4 | 23 |
| f5 | 28 |
| f6 | 27 |
| f7 | 31 |

CSF samples

| ID | age |
| --- | --- |
| f1 | 25 |
| f2 | 28 |
| f3 | 49 |
| f4 | 28 |
| f5 | 24 |
| f6 | 55 |
| f7 | 33 |
| m1 | 39 |
| m2 | 28 |
| m3 | 24 |
| m4 | 26 |
| m5 | 24 |
| m6 | 49 |
| m7 | 47 |
