# Supplementary figures and images for: Correction: A Proteomic Analysis of Individual and Gender Variations in Normal Human Urine and Cerebrospinal Fluid Using iTRAQ Quantification
Source: PLoS One. 2019 Apr 1;14(4):e0213213. doi: 10.1371/journal.pone.0213213 (PMC6443154; doi:10.1371/journal.pone.0213213)

S4 Fig

A


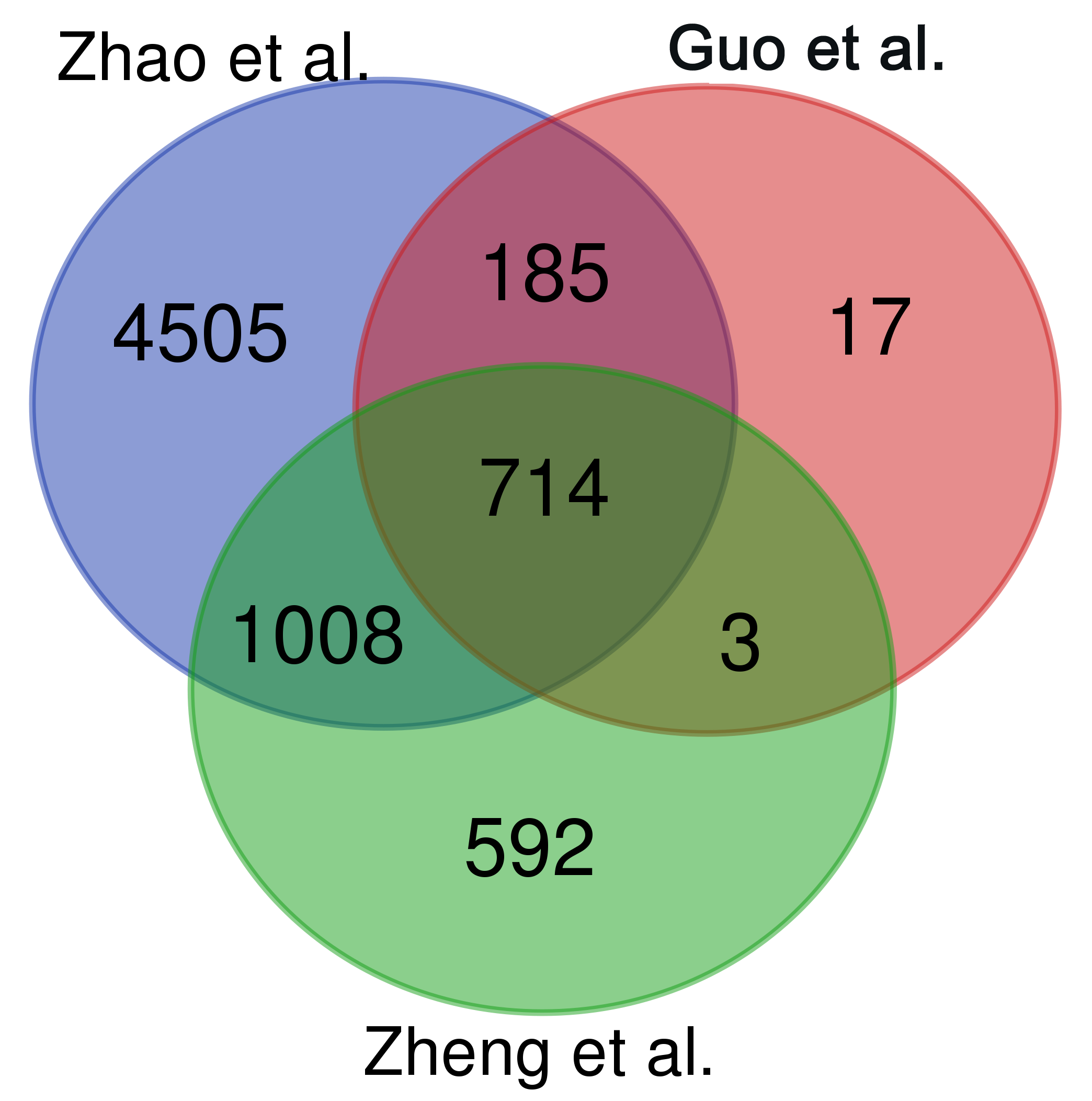


B

Supplement: S4 Fig — A: Venn diagram of the identified proteins in our dataset and other publications. B: Comparison of the normalized spectral counts for most abundant proteins and in our dataset and in other publication. (DOCX) [file pone.0213213.s005.docx]
